# 

**Published:** 1860-01

**Authors:** 


CONTENTS

OF THE

### Chicago Medical Journal for January, 1860.

ORIGINAL COMMUNICATIONS. PAGE.

ARTICLE 1.—Case of Lymphatic Tumor—Varix of the Lymphatic Trunk. By D. Brainard, M. D., (illustrated.)........ 1
ARTICLE 2.—Diphtheria. By W. G. Dyas, M. D., ........ 5
ARTICLE 3.—Removal of seven inches of Shaft of the Fibula—Recovery of the Patient—Description of a new instrument for facilitating the passage of the chain-saw under the bone. By E. S. Cooper, A. M., M. D., ........ 22
ARTICLE 4.—Operations for the restoration of the Nose. (Illustrated.). ........ 25
ARTICLE 5.—Opium: Its Influence in Febrile, Inflammatory, and other Diseases. A Prize Essay. By A. S. Hudson, M. D. ........ 27
ARTICLE 6.—Mutulation of a Child by a Midwife. By H. Carpenter, M. D. ........ 48

SELECTIONS.

Lunatics in New York City, ........ 50
Needle for Wire Suture. By R. J. Levis, M. D. ........ 51
Mecurial Stomatites, ........ 52

EDITORIAL.

Southern Students leaving the Medical Schools of Philadelphia, ........ 53
To the Medical Profession, ........ 54

SURGICAL NOTES.

1. Dislocation of the Femur of 39 days standing reduced by manipulation, ........ 54
2. Dislocation of the Hip into the Thyroid Foramen. Reduction by the method of the author, 55
3. Silver Sutures, ........ 55
4. Treatment of Indolent Ulcers by Vapor of Iodine, ........ 56

